# Upper Sternal Cleft Associated With Congenital Cardiac Defect: Single Stage Surgical Correction

**DOI:** 10.1093/icvts/ivag095

**Published:** 2026-04-07

**Authors:** Pavol Omanik, Pavel Valentik, Matej Nosal, Igor Beder

**Affiliations:** Pediatric Surgery Department, National Institute of Children’s Diseases, Comenius University in Bratislava Faculty of Medicine, 833 40 Bratislava, Slovakia; Pediatric Cardiac Centre - National Institute of Cardiovascular Diseases, Slovak Medical University, 833 48 Bratislava, Slovakia; Pediatric Cardiac Centre - National Institute of Cardiovascular Diseases, Slovak Medical University, 833 48 Bratislava, Slovakia; Pediatric Surgery Department, National Institute of Children’s Diseases, Comenius University in Bratislava Faculty of Medicine, 833 40 Bratislava, Slovakia

**Keywords:** sternal cleft, congenital cardiac defect, primary reconstruction, chest wall

## Abstract

Sternal cleft is a rare congenital malformation of anterior chest wall resulting from the failure of mesodermal migration to the midline during early embryological development. It may occur as an isolated anomaly or in connection with other congenital defects, most commonly of the cardiovascular system. The authors report the case of a 6-month-old girl with an upper sternal cleft combined with ventricular and atrial septal defects, who underwent single-stage surgical correction. The cosmetic and functional outcomes of the reconstruction were very good, with a stable anterior chest wall. The child is thriving, without cardiac limitations.

## INTRODUCTION

Sternal clefts (SC) are rare birth defects occurring in approximately 1 in 100 000 newborns and accounting for less than 0.15% of all anterior chest wall deformities.^1^ SC can be either complete or partial, depending on the presence of a connection between the 2 sternal bars. Partial defects are divided into superior or inferior, the former being more common and usually occurring as an isolated anomaly.^2^ Inferior partial defects are rare and often associated with more severe developmental anomalies such as ectopia cordis or Cantrell pentalogy.^3^ Although neonates are usually asymptomatic at birth, respiratory symptoms associated with paradoxical anterior chest wall movement may develop later in life. Surgical intervention is primarily indicated to protect intrathoracic structures from potential injury, to prevent recurrent respiratory infections and for aesthetic reasons.^2^

## CASE REPORT

We report the case of the female infant, born full-term with a birth weight of 3320 g, and adequate postpartum adaptation. After birth, paradoxical movement of the anterior chest wall was observed at the site of the missing sternum, with visible pulsation of the heart, covered by a thin skin-pericardial membrane. A supraumbilical raphe was noted along the midline. The patient was cardiorespiratory compensated, without clinical symptoms. Postnatal CT confirmed 2 ossified bases of the fifth sternal segment and the xiphoid process in the midline. The remaining ossified remnants were displaced laterally (maximum 36 mm), with lateralization of the medial clavicular ends. The midline chest defect was in close contact with the thymus, the ventral edge of the truncus pulmonalis and the right ventricle (Video 1). Echocardiography revealed a subaortic ventricular septal defect (3.5 mm) and an atrial septal defect (ostium secundum), with a haemodynamically significant left-to-right shunt. The multidisciplinary team scheduled surgery at 6 months of age to balance cardiological, cardiosurgical, and pediatric surgical considerations, maximizing overall clinical benefit while minimizing associated risks.

The operation (Video 2) started with a longitudinal midline incision extending from the jugulum to the supraumbilical region. The skin and subcutaneous tissue were carefully dissected from the closely attached pericardium. The pectoral muscles were detached from their insertions on the anterior chest wall, exposing the full course of both sternal bars, adjacent portions of the clavicles and ribs. A cranial SC was identified; the caudal part of the sternum was intact. A V-shaped sternotomy of the caudal fused section was performed to facilitate subsequent approximation of the sternal bars. This was followed by complete thymectomy, pericardiotomy, clipping of the ligamentum arteriosum, and initiation of extracorporeal circulation. The ventricular septal defect was closed using interrupted substantiated stiches; the ostium secundum, with a purse-string (“tobacco pouch”) suture, via the right atrium. One substernal drain and a Gore-Tex membrane were placed along the pericardial margin. Next, longitudinal perichondrial incisions were made along both sternal bars, and the perichondrial flaps were mobilized medially, yielding 2 flaps approximately 2 × 0.75 cm. To expand chest volume and increase the effective rib length, Z-shape chondrotomies were performed bilaterally along the mid-clavicular lines from the second to the fifth ribs. 2–0 non-absorbable sutures were placed behind the sternal bars, and 4–0 absorbable sutures on the mobilized perichondrial flaps. Temporary juxtaposition of the sternal bars was performed by crossing over the non-absorbable sutures under close haemodynamic and respiratory monitoring. As the patient remained stable, the sutures were tied, approximating the sternal bars. The operation was completed by re-approximation of the pectoral muscles over the “neo-sternum”, placement of a submuscular drain, and surgical wound closure.

The child required mechanical ventilation for 3 days postoperatively. The postoperative course was uneventful, and the patient was discharged after 10 days. At 1-year follow-up, the anterior chest wall was stable, with good respiratory biomechanics and a good cosmetic result. The caudal portion of the “neo-sternum” was minimally prominent, resembling a pectus carinatum. Lateral radiography showed 3 sternal ossification centres in formation.

## DISCUSSION

The diagnosis of SC is usually made postnatally, based on the presence of paradoxical midline movement, though it can be detected prenatally by ultrasonography.^4^ Postnatal CT is valuable for determining the type and size of the cleft, its relationship to the heart, and for planning surgical repair.^4^

The success of surgical correction depends on the patient’s age and the cardiovascular system’s haemodynamic response to the new anterior chest wall configuration after surgery. In general, primary closure is recommended in children younger than 3-4 months of age.^5^ At this stage, the chest is most flexible, minimizing compression of the underlying mediastinal structures after reconstruction and improving postoperative compliance and cosmetic results.^4^ Also, primary closure is believed to provide space for continued normal sternal development.^2^

Perichondrial flaps prepared from the sternal bars, together with cranial ribs chondrotomies, are standard techniques for obtaining additional thoracic volume during reconstruction and are well described in the literature.^2^^,^^4^^,^^5^ If these steps are not sufficient, thymectomy or clavicular dislocation may be considered.^4^

In cases of unfavourable haemodynamic response, the use of additive autologous or synthetic material may be necessary for successful reconstruction. Similar challenges occur in older children when the elasticity of the anterior chest wall is reduced.^3^ Biological substitutes, such as bone graft interposition (costal cartilage, tibial graft), muscle flap interposition, or synthetic patches (Marlex, Teflon), may be valuable alternatives.^2^^,^^5^

## Data Availability

The data underlying this article are available in the article and in its online supplementary material.
